# Design of a lightweight recognition network for adult locusts and grasshoppers based on deep learning

**DOI:** 10.1016/j.isci.2025.113096

**Published:** 2025-07-10

**Authors:** Youchen Zhen, Haibin Han, Hongru Yue, Yanmin Shan, Wei Wu, Ning Wang, Yanyan Li

**Affiliations:** 1 Research Center for Grassland Entomology, Inner Mongolia Agricultural University, Hohhot 010020, China; 2 Key Laboratory of Biohazard Monitoring, Green Prevention and Control for Artificial Grassland, Ministry of Agriculture and Rural Affairs, Institute of Grassland Research of Chinese Academy of Agricultural Sciences, Hohhot 010010, China; 3 Key Laboratory of Agricultural Blockchain Application, Ministry of Agriculture and Rural Affairs & Agricultural Information Institute, Chinese Academy of Agricultural Sciences, Beijing 100081, China; 4 Inner Mongolia Forestry and Grassland Pest Control and Quarantine Station, Hohhot 010020, China

**Keywords:** environmental science, agricultural science, artificial intelligence

## Abstract

Grassland locusts and grasshoppers play a vital role in driving the dynamic changes of grassland ecosystems. In this study, we propose a lightweight deep learning-based network model for accurate identification of locust and grasshopper genera. Two image datasets were constructed, each containing 60 genera of locusts and grasshoppers. To improve recognition accuracy and computational efficiency while reducing floating-point operations (FLOPs) and the number of parameters, we introduced the channel-wise principal-component attention (CPCA) attention mechanism module and replaced part of the EfficientNet convolution modules with GhostConv, which incorporates the efficient channel attention (ECA) attention mechanism, thereby developing the CGENet model. During training, transfer learning and the Adam optimization algorithm were employed, significantly enhancing accuracy. This study makes precise control of locusts and grasshoppers feasible, thereby helping to reduce the damage they cause to agricultural production.

## Highlights

• The study constructs large and small datasets covering 60 locust and grasshopper genera
• CGENet integrates lightweight modules with multi-attention mechanisms
• The model demonstrates superior accuracy across multiple benchmark datasets
• The system delivers excellent deployment performance on edge devices

## Graphical abstract

**Figure:**
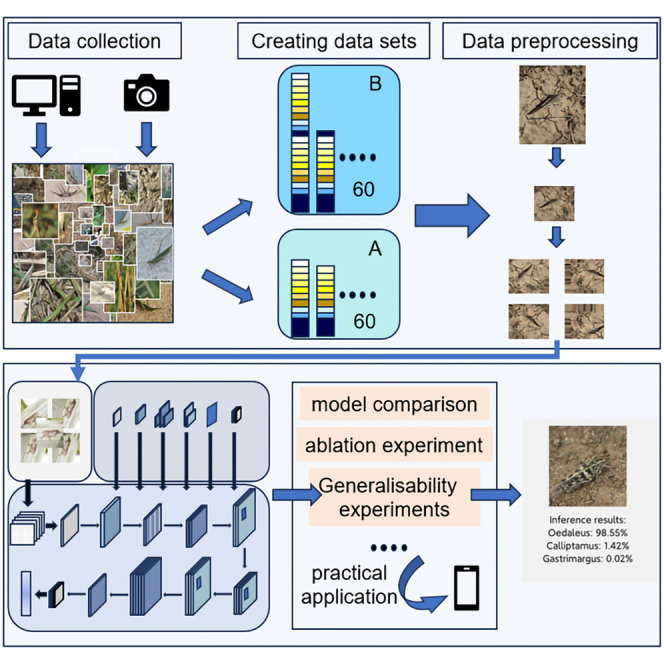

## Introduction

Locust and grasshopper plague is one of the disasters that seriously affect agriculture and animal husbandry, and its scope is expanding due to climate change.^1^ The invasion of locusts and grasshoppers leads to the destruction of farmland and pastures, posing challenges for farmers in meeting market and family demands. This, in turn, impacts food supply stability and safety.^2^ Furthermore, the decline in food production resulting from the locust and grasshopper disaster has repercussions on international food trade, while potential increase in food prices within affected regions hinder economic development.^3^ In South Africa, for example, four different species of locusts and grasshoppers have caused widespread destruction to crops and economies at various times.^4^ In the ecological environment, locusts and grasshoppers possess significant destructive potential, prompting the utilization of pesticides and other chemical control measures for their eradication. However, it is crucial to acknowledge that the extermination of locusts and grasshoppers also exerts a certain impact on the ecology.^5^ Therefore, monitoring and identifying locust and grasshopper populations serve as meaningful endeavors.

In recent years, deep learning has continued to evolve while acting in all directions of agriculture and also on locust and grasshopper recognition. The evolution of classification networks has been pivotal in this transformation.^6^ Notably, The AlexNet, proposed by Krizhevsky et al., marks a significant milestone in the field of deep learning, characterized by its depth, hierarchical architecture, parallelism, and non-linearity.^7^ Zhu et al. utilized these characteristics to analyze a dataset consisting of 22 species of Lepidoptera, achieving a notable classification accuracy exceeding 98.57%.^8^ Furthermore, Simonyan et al. developed the VGGNet architecture, making a significant contribution to the field.^9^ Kusrini et al. exploited the advantages of VGGNet, including its simple structure, ease of implementation, and high performance in image recognition, to enhance a dataset of 15 pest species, achieving an accuracy of 76%.^10^ While this accuracy is impressive, it highlights the potential for further improvement, especially when compared to the results obtained with AlexNet. Over time, He et al. addressed critical challenges in deep network training—gradient disappearance and gradient explosion—with the introduction of ResNet.^11^ Zhang et al. leveraged the deeper and more efficient advantages of ResNet-50, incorporating the coordinated attention (CA) attention mechanism module for further enhancement, and evaluated its performance on the AppleLeaf9 dataset. The model achieved an accuracy of 98.32%, demonstrating the effectiveness of combining deep learning architectures with attention mechanisms for pest detection tasks.^12^ In the field of locust recognition, although the ResNet-Locust-BN model proposed by Ye et al.^13^ in 2020 innovatively adopted the ResNet50 architecture to achieve high-precision identification of locust species and growth stages (with an accuracy rate of 90.16%), significantly outperforming traditional models such as AlexNet and GoogLeNet, its complex network structure resulted in high computational resource requirements. Similarly, while the improved ResNet50-based model developed by Jayandhi’s team^14^ in 2021 further enhanced classification performance and provided technical support for locust plague early warning.

While these models demonstrate considerable accuracy, their substantial size remains a limitation. The transition toward lightweight yet high-precision deep learning models has become crucial for deployment in resource-constrained environments. Several architectures have recently emerged that successfully balance model size, accuracy, and computational efficiency, finding applications across multiple domains. SqueezeNet was designed to be compact and efficient. It reduced the model size and the number of parameters, making it suitable for environments with limited resources. Additionally, SqueezeNet enables fast reasoning, allowing for quick decision-making in a short time frame.^15^ Huang et al. enhanced SqueezeNet for identifying Pine Wood Nematode disease, achieving an accuracy of 94.90%.^16^ MobileNet is a lightweight deep learning model architecture suitable for mobile devices and embedded systems with lower compute and storage requirements. It maintains high accuracy while having smaller model sizes and lower computational and storage requirements.^17^ Bi et al. used MobileNet for leaf disease identification of apple tree, achieving an accuracy rate of 73.50%.^18^ ShuffleNet is another efficient network architecture that balances high accuracy with low computational complexity.^19^ Feng et al. improved ShuffleNet for potato late blight detection by introducing an attention module and optimizing the model structure. The enhanced model achieved a classification accuracy of 94% on embedded devices.^20^ EfficientNet stands out for its efficient performance combined with network structure search methods for better model scaling and parameter optimization.^21^ Zhang et al. (2022) proposed using EfficientNet and a refined version of the Class Activation Mapping algorithm for maize pest identification. The model achieved an accuracy of 93.85% with a model size 90% and 85% lower than ResNet101 and DenseNet161, respectively.^22^ In terms of research on the lightweight use of locust models, Qiu et al.^23^ (2024) developed a YOLOv7-MobileNetV3-CA model that combines a lightweight MobileNetV3 backbone with CA to achieve 27% model compression and 4.48% AP improvement while maintaining detection performance. However, current approaches still face challenges in achieving optimal accuracy and sufficiently lightweight edge deployment. This research aims to address these limitations by developing improved lightweight architectures that simultaneously improve accuracy and reduce computational complexity.

These architectures demonstrate the feasibility of developing lightweight yet high-precision models suitable for resource-constrained applications, including locust and grasshopper recognition. Leveraging advancements in model design and optimization techniques, researchers can now develop effective solutions to address complex real-world challenges. Controlling the scale and accuracy of the model poses the greatest challenge in these studies, as reducing the model’s scale inevitably compromises its accuracy to some extent.^24^ As a result, striking the right balance between scale and accuracy becomes a daunting task for researchers.

To address these challenges, the main contributions of this article are as follows.

(1) The study compiled a dataset that includes various locust and grasshopper genera after a process of collection and curation.
(2) The study employed various neural networks to train the locust and grasshopper genera dataset and conducted a comparison among them.
(3) The study replaced model components and refined the structure to make it more lightweight and accurate.

## Results

### Comparison of different models

**Figure 1:**
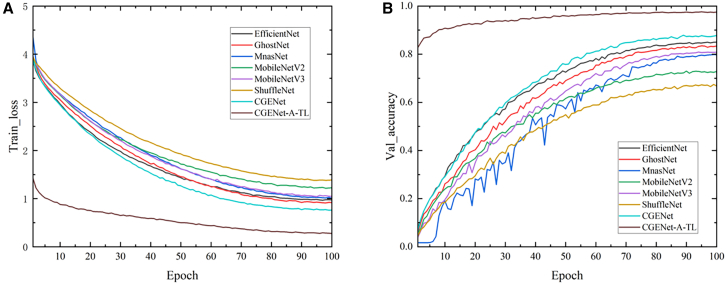
Comparison results of different models (A) shows the training loss rate between individual networks; (B) shows the accuracy rate between individual networks. There is a significant decrease in the loss rate and a large increase in the accuracy of our networks with different training strategies.

The improved network was compared with other lightweight models, including GhostNet, MnasNet, MobileNetV2, MobileNetV3, ShuffleNet, and the original EfficientNet model, using dataset A. The comparison focuses on the training loss rate and validation accuracy performance in the context of locust and grasshopper recognition task. To enhance the model’s performance, several adjustments were made to the training strategy. Firstly, dataset B was utilized as a starting point for transfer learning, leveraging the knowledge learned from a related task to improve the model’s performance on the locust and grasshopper recognition task. Secondly, the optimization algorithm was switched from stochastic gradient descent (SGD) to Adam (adaptive moment estimation), which is known for its adaptive learning rate and often results in faster convergence. Finally, CosineAnnealingLR was applied as the learning rate decay algorithm, which gradually decreases the learning rate following a cosine decay schedule, helping the model to converge more stably. The results of these comparisons and adjustments are presented in Figure 1.

As shown in the figure, the training loss curves of all models gradually decrease and eventually stabilize as training progresses. This indicates that the learning rate settings are reasonable and that the models have undergone sufficient training. In terms of loss reduction, CGENet and GhostNet experienced the fastest decrease, outperforming other models and demonstrating excellent fitting performance. As shown in Figure 1B, in terms of validation accuracy, our model and the original EfficientNet model achieved the best performance, both exceeding 0.85. In contrast, ShuffleNet’s performance was relatively poor, potentially due to insufficient model depth during training. Finally, with the aid of transfer learning and the Adam optimizer, our model showed significant improvements. Specially, the training loss decreased to 0.28, and the validation accuracy increased to 0.97. This demonstrates that our training strategy is well-suited for training our model.

The results indicate that GhostNet and EfficientNet exhibit superior performance and are suitable for the application in this study. Upon comparing the training processes of each model, it is evident that the improved model demonstrated outstanding superiority in both training loss and validation accuracy.

### Classification performance of the model

To validate the performance advantages of the improved model, we conducted a comparative performance analysis of multiple models on the test set from the dataset. As shown in Table 1, when trained using the same strategy, CGENet achieved the highest Prec of 89.76%. This is 0.04%, 0.076%, 0.138%, 0.069%, 0.229%, and 0.027% higher than GhostNet, MnasNet, MobileNetV2, MobileNetV3, ShuffleNet, and EfficientNet, respectively. This significant difference demonstrates that CGENet has a clear advantage in the locust and grasshopper genus identification task. In addition to precision, CGENet also showed the highest values for accuracy (Acc), recall (Rec), and F1 score, as shown in Table 1. Specifically, CGENet achieved an accuracy of 99.66%, a recall of 89.73%, and an F1 score of 89.66%. These values are 0.65%, 19.85%, and 19.96% higher than the lowest values observed in ShuffleNet, respectively. Of course, as a lightweight model, the number of floating-point operations (FLOPs) and parameters (Params) of the model are also crucial factors to consider. These metrics represent the model size and computational complexity. According to Table 1, ShuffleNet has the lowest FLOPs and Params, which may result in insufficient computational power and therefore poorer performance. In contrast, CGENet has relatively fewer parameters and a smaller size compared to the original EfficientNet model, with 0.14G and 1.26M fewer parameters, respectively. This significantly reduces the computational and memory requirements while improving the model’s performance across various metrics.

**Table 1:** Evaluation index results of different models

Model	Prec (%)	Rec (%)	Acc (%)	F1 (%)	FLOPs (G)	Params (M)
EfficientNet	87.06	87.01	99.57	86.90	0.54	4.08
GhostNet	85.76	85.68	99.52	85.58	0.20	3.97
MnasNet	82.19	82.16	99.41	82.02	0.44	3.19
MobileNetV2	76.04	75.91	99.20	75.68	0.43	2.30
MobileNetV3	82.87	82.86	99.43	82.70	0.30	4.28
ShuffleNet	69.91	70.15	99.01	69.70	0.20	1.33
CGENet	89.76	89.73	99.66	89.66	0.40	2.82
CGENet +A+TL	97.55	97.52	99.92	97.52	0.40	2.82

The table shows the detailed performance of each model in the assessment indicators.

Finally, by modifying the training strategy to improve accuracy, we were able to significantly enhance the model’s Prec to 97.55%. Therefore, based on all evaluation metrics, CGENet is undoubtedly an excellent performing model.

#### Confusion matrix analysis

**Figure 2:**
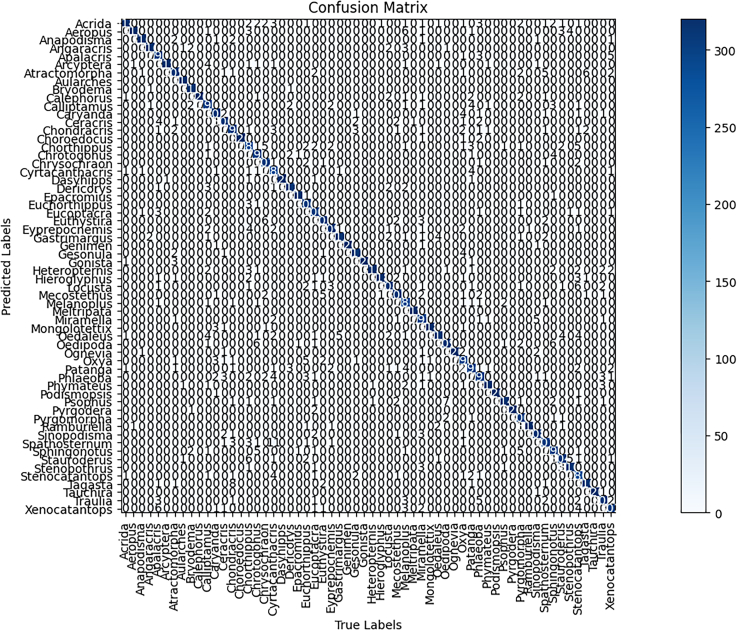
Confusion matrix diagram The figure shows a mixed matrix representation of 60 locust and grasshopper genera, reflecting the model’s excellence with its dark diagonal lines.

To further analyze the performance of CGENet, we present a confusion matrix in Figure 2, which display the classification results of the 60 locust and grasshopper genera in the CGENet-A-TL model. A confusion matrix is a tool that visually represents the performance of a classification model by displaying the relationship between the predicted and actual results in the form of a matrix.^25^ The rows and columns of the matrix represent the predicted and actual categories of the locust and grasshopper genera, respectively. Each cell in the matrix indicates the number of occurrences of a pairing between the actual and predicted categories. The shading of the cells indicates the magnitude of the values, with darker colors representing higher numbers.

In terms of performance, the stability of the model’s predictions can be inferred from the distribution of numbers and shading of cells in the confusion matrix, excluding the diagonal cells. A model with stable predictions will have fewer dispersed numbers and darker colors in the off-diagonal cells. As shown in Figure 2, the diagonal cells are deep dark, indicating that the CGENet-A-TL model makes accurate predictions for the 60 locust and grasshopper genera. Additionally, most of the cells outside the diagonal are zero, with no darker colors present, further confirming that the model has high recognition accuracy and stability for the locust and grasshopper genera. However, it is worth noting that some categories still experience errors, suggesting that these particular categories may be more difficult to identify than others.

#### Visualization analysis

**Figure 3:**
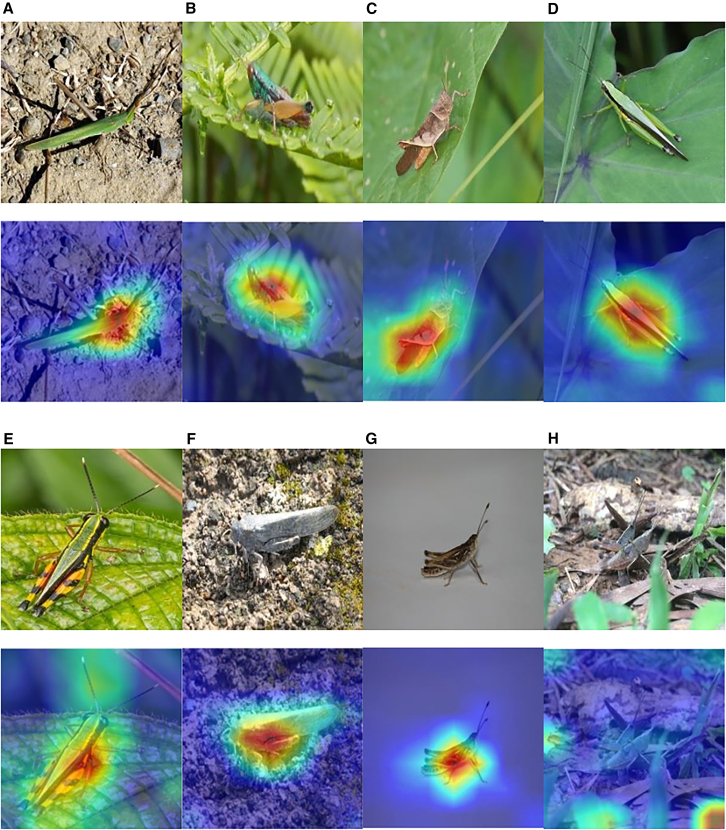
Confusion matrix diagram (A–H) Grad-CAM visualizations of eight locust and grasshopper species, (A–G) all perform very well, but (H) does not have the correct recognition features.

In this study, we applied gradient-weighted class activation mapping (Grad-CAM) to CGENet to further conduct qualitative analysis. Grad-CAM is a technique used to visualize and understand the decisions made by convolutional neural networks (CNNs). By analyzing the gradients in the convolutional layers, it decodes the importance of each feature map for a specific class and generates a heatmap. Through this heatmap visualization, we can clearly see the important areas of the input image that contribute to predicting a specific class.^26^ As shown in Figure 3 this heatmap visualization represents 8 locust and grasshopper genera in the dataset. The images are obtained from the visualization of the last convolutional layer of the model, where the top image is the original image, and the bottom part shows the corresponding heatmap. From image (A) to image (G), it can be observed that the areas of interest identified by the model accurately cover the locust and grasshopper individuals in the images.

Additionally, as shown in image (E), the model’s areas of interest not only cover the body of the locusts and grasshoppers but also extend to the antennae. Compared to the other images, this indicates that the model extracts different features for different categories, enhancing its accuracy. However, in some images, the model’s attention is not focused on the target object, as shown in image (H). In this case, the prominent area in the heatmap is not located on the locusts and grasshoppers but on the edges of the image, resulting in a recognition accuracy of only 33.65%. Upon analyzing the original image, it is observed that the object consists of two overlapping locusts and grasshoppers, and there is occlusion in the image, both of which affects feature extraction and reduce recognition accuracy.

Overall, despite the unavoidable factors present in the images, CGENet demonstrates excellent performance in extracting object features.

### Ablation experiments

Table 2 presents the comparative performance results of various enhanced models based on the EfficientNet architecture in ablation studies. This research systematically evaluates the impact mechanisms of different modules including CPCA (channel and position-wise convolutional attention), GhostConv (lightweight convolution), and ECA (efficient channel attention) on model performance. Experimental results demonstrate that the EfficientNet+CPCA model, incorporating the CPCA module, achieves superior classification performance with precision, recall, and F1-score reaching 90.56%, 90.47%, and 90.45% respectively, showing significant improvement over the baseline model. However, this performance enhancement comes with increased computational costs, as the model’s FLOPs and Params rise to 0.62G and 3.62M correspondingly. In terms of model efficiency, the EfficientNet+GhostConv variant demonstrates a favorable balance, maintaining 88.74% precision and 88.65% F1-score while keeping computational complexity and parameter count at only 0.29G and 2.82M, respectively. Notably, the EfficientNet+GhostConv (ECA) variant achieves more balanced performance metrics (89.39% precision, 89.31% recall, and 89.26% F1-score) while preserving the same computational budget (0.29G FLOPs, 2.82M Params). Comprehensive analysis indicates that the CGENet model achieves the optimal balance between performance and complexity. Despite its relatively higher computational requirements (0.40G FLOPs) and parameter count (2.82M), this model delivers outstanding performance across all evaluation metrics (89.76% precision, 89.73% recall, and 89.66% F1-score), demonstrating robust overall competitiveness in the comparative study.

**Table 2:** Results of ablation experiment

Model	Prec (%)	Rec (%)	Acc (%)	F1 (%)	FLOPs (G)	Params (M)
EfficientNet	87.06	87.01	99.57	86.90	0.54	4.08
EfficientNet+CPCA	90.56	90.47	99.65	90.45	0.62	3.62
EfficientNet+ GhostConv	88.74	88.72	99.62	88.65	0.29	2.82
EfficientNet+ GhostConv(ECA)	89.39	89.31	99.64	89.26	0.29	2.82
EfficientNet+ GhostConv +CPCA	88.99	88.93	99.63	88.87	0.39	2.83
CGENet	89.76	89.73	99.66	89.66	0.40	2.82
CGENet+A	91.98	91.91	99.73	91.89	0.40	2.82
CGENet+A+TL	97.55	97.52	99.92	97.52	0.40	2.82

In the table, EfficientNet is the original model; EfficientNet+CPCA is the addition of the CPCA attention module; EfficientNet+ GhostConv is the change of some of the convolution modules in the model to GhostConv; GhostConv(ECA) is the change of the SE modules in GhostConv to ECA module; CGENet is the combination of EfficientNet+ GhostConv(ECA)+CPCA; A denotes a change in modeling strategy from SGD to Adam; TL denotes the use of transfer learning.

From the perspective of the training strategy, Adam computes a separate learning rate for each parameter by combining estimates of the first moment (mean) and the second moment (variance) of the gradients. This allows different parameters to dynamically adjust their learning rates based on their own update history, whereas SGD uses a fixed or gradually decaying learning rate. As a result, the model’s evaluation metrics improved, with Prec, Rec, Acc, and F1 increasing by 2.22%, 2.18%, 0.07%, and 2.23%, respectively. This indicates that the Adam optimizer is more suitable for locust and grasshopper genus recognition than SGD. Furthermore, the introduction of transfer learning significantly enhanced the performance of CGENet, with precision sharply increasing to 97.55%, representing a 5.61% improvement. This suggests that transfer learning based on dataset B is highly effective for model training.

#### Generalization performance of CGENet

To demonstrate the generalizability of CGENet, we sought to evaluate its performance on three additional publicly available classical datasets. The first dataset is IP102,^27^ which comprises over 75,000 images representing 102 distinct categories of insect pests. As one of the most comprehensive datasets for insect pest recognition, IP102 captures a wide variety of pest forms, including eggs, larvae, and adults. This extensive coverage endows the dataset with high diversity and broad applicability. The second dataset is PlantDoc,^28^ a visual dataset for plant disease detection, consisting of 2,598 images across 13 plant species and 17 disease categories. Despite its relatively small size, the dataset features a high diversity of categories, making it an ideal benchmark to evaluate model performance in scenarios with limited data. The third dataset is a locust dataset named GLCD, which contains a total of 4,454 high-resolution images of locusts in six categories.

CGENet and other lightweight models were evaluated on three distinct datasets with different data partitioning schemes: the first two datasets were split into training, validation, and test sets in a 7:2:1 ratio, while the third dataset followed an 80% training, 10% validation, and 10% test configuration. As shown in Table 3, despite potential limitations arising from dataset complexity and relatively small sample sizes per category, CGENet consistently outperformed all competing models across all evaluation metrics. Specifically, it achieved superior accuracy scores of 59.00%, 44.11%, and 85.43% on the respective datasets, accompanied by corresponding F1-scores of 56.95%, 43.37%, and 84.36%. These robust experimental results demonstrate CGENet’s exceptional generalization capability and stable performance across datasets of varying scales.

**Table 3:** Comparative structure of the model in the three datasets

Model	IP102		PlantDoc		GLCD	
	Prec (%)	F1 (%)	Prec (%)	F1 (%)	Prec (%)	F1 (%)
EfficientNet	53.98	49.74	38.75	35.26	84.27	83.17
GhostNet	58.85	56.17	34.87	33.12	71.58	71.41
MnasNet	58.72	55.86	37.82	37.23	80.01	80.16
MobileNetV2	55.58	51.37	36.51	34.93	77.69	76.47
MobileNetV3	52.84	49.95	42.94	40.24	82.70	82.39
ShuffleNet	54.68	52.30	35.62	34.27	75.40	75.19
CGENet	59.00	56.95	44.11	43.37	85.43	84.36

The table shows the performance results of each model on the three datasets IP102, PlantDoc and GLCD. CGENet outperforms all other models, achieving a precision that is 0.15% higher than the second-best model on IP102 and 0.78% higher in terms of F1-score. On the PlantDoc dataset, CGENet’s precision is 1.17% higher than the second-best model, while its F1-score is 3.13% higher. On the GLCD dataset, CGENet outperformed the second-ranked model by 1.16% in terms of accuracy and by 1.19% in terms of F1 score.

#### Validation and statistical significance

To systematically validate the performance superiority of CGENet over comparative models and ensure the robustness of our architectural improvements, we performed extensive 5-fold cross-validation coupled with statistical significance testing comparing the original and enhanced model variants.

**Figure 4:**
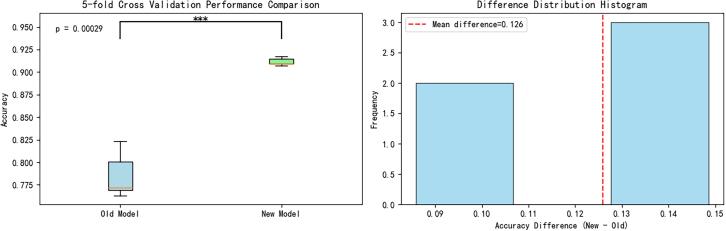
Comparison of 5-fold cross-validation performance and distribution of accuracy difference between old and improved models The figure presents comparative analysis results between the improved and original models from five independent experimental replicates. The left panel displays 5-fold cross-validation results (data are represented as mean ± SEM; ∗∗∗*p* < 0.001). The right panel shows the difference distribution histogram, with the mean difference indicated by a red dashed line.

Figure 4 presents a comparative analysis of the performance between the improved and baseline models using 5-fold cross-validation. The boxplot (left panel) demonstrates that the improved model not only achieves higher overall accuracy than the baseline model but also exhibits a more concentrated distribution, indicating superior stability. Statistical analysis reveals a highly significant difference between the two models (*p* = 0.00029, ∗∗∗ denotes *p* < 0.001).

The histogram (right panel) clearly illustrates the distribution characteristics of accuracy differences, with the red dashed line indicating a mean difference of 0.126. Notably, the majority of differential values are concentrated within the 0.12–0.15 range, further substantiating the consistent performance advantage of the improved model. Collectively, these results demonstrate that the improved model significantly outperforms the baseline model in terms of both accuracy and stability.

### Practical application of the model

**Figure 5:**
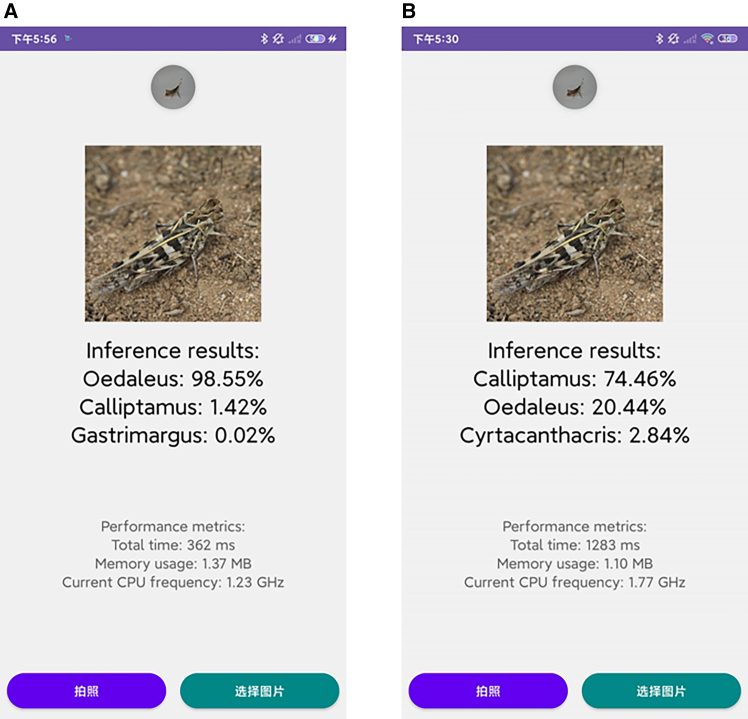
Comparison of actual test results on mobile (A) Shows the results of the CGENet mobile test and (B) shows the results of the original EfficientNet mobile test.

To validate the practical deployment performance of our model, we implemented both CGENet and the original EfficientNet as mobile applications using the Android Studio framework. The evaluation was conducted on a smartphone (Android 10, 6GB RAM, Qualcomm Snapdragon 845 processor), with the comparative results presented in Figure 5.

Experimental results demonstrate that the CGENet-based application successfully achieved precise locust and grasshopper genus recognition with an accuracy of 98.55%, while the original EfficientNet-based implementation failed to identify the locust and grasshopper genus in test images. In terms of computational efficiency, CGENet exhibited significant improvements across multiple metrics: the inference time was reduced from 1283 ms to 362 ms (a 71.8% reduction), CPU frequency usage decreased from 1.77 GHz to 1.23 GHz (30.5% lower), with only a marginal 0.27 MB increase in memory consumption (from 1.10 MB to 1.37 MB). These comprehensive metrics clearly demonstrate the superior performance of our improved model in real-world deployment scenarios.

## Discussion

### The impact of module substitution position

**Figure 6:**
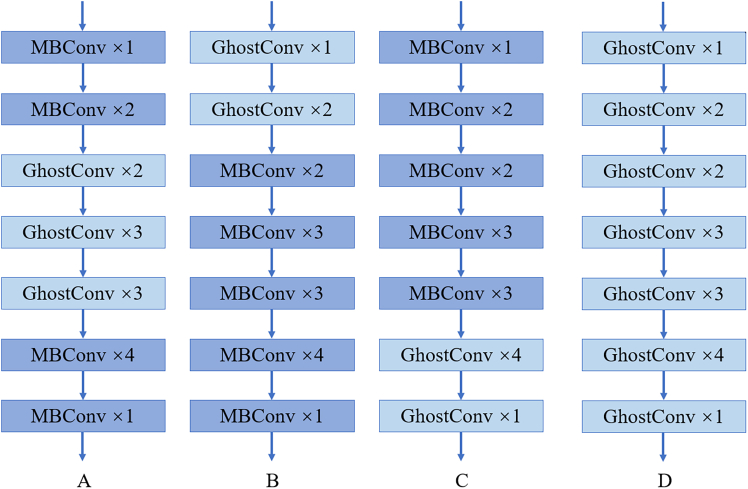
Structure of different substitute module position models (A) refers to replacing the middle three parts of MBConv, (B) refers to replacing the first two parts of MBConv, (C) refers to replacing the last two parts of MBConv, and (D) refers to replacing the entire structure of MBConv.

To investigate the balance of model improvements, the study analyzes and compares the positions where the convolution modules were replaced. Specifically, we replaced different parts of the model’s main convolution blocks, as illustrated in Figure 6. Model A represents the CGENet design with GhostConv replacing the middle three blocks, model B replaces the top two blocks, model C replaces the bottom two blocks, and model D represents a complete replacement of all blocks, resulting in a total of four cases. These models were trained on dataset 1, and the training results are shown in Table 4.

**Table 4:** Evaluation results of four different models

Model	Prec (%)	FLOPs (G)	Params (M)
A	91.98	0.40	2.82
B	89.88	0.55	3.60
C	88.82	0.46	1.04
D	64.23	0.18	0.23

The highest Prec in the table is achieved by our designed CGENet, reaching 91.98%. Models B and C follow closely with precisions of 89.99% and 88.82%, respectively. On the other hand, the model with all modules replaced, model D, showed the lowest performance with a precision of only 64.23%, which is 27.75% lower than CGENet. However, it is worth noting that model D had the smallest number of Params, only 0.23M, and its FLOPs were also minimal, at 0.18G. This likely explains its lower accuracy, as it may have lacked the capacity to learn complex patterns in the data. In comparison, CGENet, while not having the smallest number of Params, achieved high precision with relatively low FLOPs, demonstrating the superiority of our design and showing a balanced trade-off between Precision, FLOPs, and Params.

### Analysis of locust and grasshopper genus results based on different data volumes

**Figure 7:**
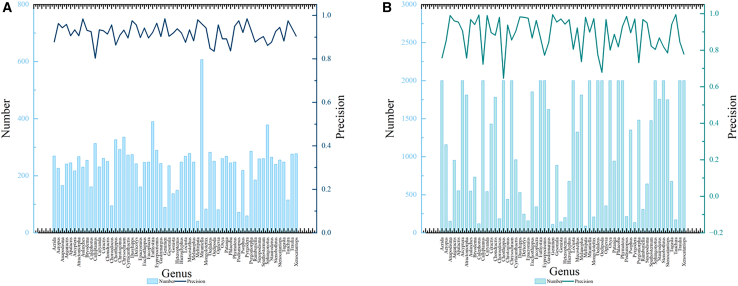
The precision results of different data volumes (A) Shows the original number and precision of each locust and grasshopper genus in dataset A, (B) shows the original number and precision of each locust and grasshopper genus in dataset B.

Furthermore, we performed a systematic analysis of the Prec for the 60 locust genera in the test sets of both datasets using the proposed CGENet. Specifically, we compared the test results under different data quantities. As shown in Figure 7, Chart A displays the Prec for most of the locust and grasshopper genera exceeds 90%. Through image analysis, it was observed that these genera have clear and distinctive data characteristics, and within these 60 genera, there is minimal similarity between them. There is also a noticeable point on the line where the precision drops indicating that the model performs poorly in recognizing these genera. The bar chart below Chart A shows the number of images for each genus, revealing that most of the original data quantities range from 100 to 300, with only a few genera having less than 100 or more than 300, indicating a relatively balanced data distribution. In contrast in Chart B, the Prec line shows significant fluctuations, with most genera achieving over 85% Prec, but the lowest dropping to 65%, indicating poor model performance for certain genera. The corresponding bar chart below Chart B reveals a large variation in the number of original images, ranging from 100 to 2000, which may suggest an imbalanced data distribution. This imbalance could potentially be a contributing factor to the model’s poor performance for certain genera.

By comparing both charts, we draw the following conclusions: (1) highly similar locust and grasshopper genera, such as *Acrida*, *Pyrgomorpha*, and *Tagasta*, tend to affect each other’s precision. These genera share similar morphological characteristics, and if the data quality is low and blurred, it leads to lower precision for all three. (2) Locust and grasshopper genera with more species tend to have lower recognition accuracy, while genera with fewer species perform better. For example, the genus *Chorthippus*, which has the lowest precision, suffers due to the large number of species included in the dataset. To address this, further subdivision of genera may be necessary. (3) Imbalanced data quantities and poor data quality, such as blurry, occluded, or noisy images, also impact the model’s performance. Moreover, manually captured images may not provide sufficient samples of certain locust and grasshopper genera.

To address the challenges of low recognition accuracy for certain locust and grasshopper genera caused by data imbalance and visual similarity, this study employed a GAN-based synthetic data augmentation approach. Specifically, we trained a generative adversarial network to produce high-quality synthetic images, which served two purposes: (1) balancing the class distribution in the training dataset, and (2) enriching the feature space with more discriminative characteristics.

**Figure 8:**
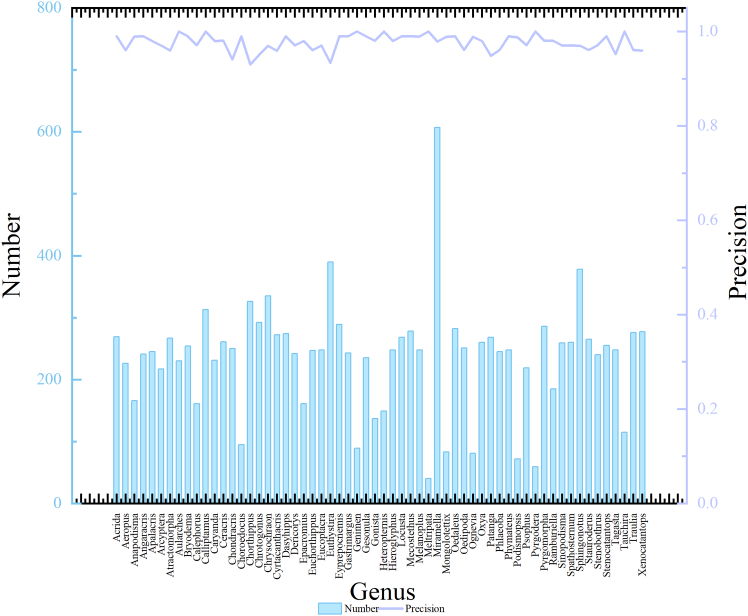
Experimental results using GAN amplification dataset

As demonstrated in Figure 8, the proposed method significantly improved the recognition performance across all 60 locust and grasshopper genera. The precision curve exhibits both higher values and smoother distribution compared to the baseline, indicating that the GAN-generated samples provide more effective feature.

### Limitations of the study

In summary, compared to the original EfficientNet, our network enhances feature extraction capabilities, resulting in improved accuracy for image recognition. Additionally, it reduces the computational load, making it suitable for devices with limited computational resources, and minimizes the model parameter size, thereby occupying less memory. However, our experiments have certain limitations. Moving forward, we aim to further balance model lightweight design and accuracy, with the goal of developing more compact and accurate models. Furthermore, more refined classification can be achieved by focusing on identifying locust and grasshopper species rather than just genera. Additionally, capturing images manually will improve the quality and comprehensiveness of the dataset. Finally, our experiments will continue, contributing to further advancements in the accuracy and intelligence of locust and grasshopper recognition.

### Conclusion

Locusts and grasshoppers have a significant impact on agriculture and livestock farming. With the development of precision agriculture, the use of lightweight devices to accurately identify and control locusts and grasshoppers is becoming increasingly important. In this study, we focused on applying and improving lightweight deep learning models to identify common locust and grasshopper genera. Specifically, we compared seven models, including our proposed model, for the classification task of 60 locust and grasshopper genera. To achieve this, we first built two datasets using images collected from various sources, including iNaturalist, GBIF, Baidu, Bing, Google, and offline sources. Dataset A contains 14,156 raw images, while dataset B contains 68,807 raw images. We also implemented data augmentation was using Keras to enhance the size and diversity of our datasets.

We conducted a comparative analysis of the performance of seven models, including GhostNet, MnasNet, MobileNetV2, MobileNetV3, ShuffleNet, EfficientNet, and our proposed CGENet, on dataset A. We discussed the balance of different improvements and the limitations of the models on both datasets. The results showed that, when using transfer learning on dataset B to train dataset A, and with the Adam optimization algorithm, CGENet achieved the best results with a Prec of 97.55%, FLOPs of 0.40G, and Params of only 2.82M.

In summary, this study successfully constructed two high-quality image datasets comprising 60 locust and grasshopper species, and systematically compared the performance of various lightweight deep learning models. Innovatively, we proposed an improved lightweight CGENet model, with experimental results demonstrating its significant superiority over existing methods in both recognition accuracy and computational efficiency. Notably, lightweight networks exhibit unique practical value in agricultural intelligent applications: their characteristics of low computational resource requirements and high inference efficiency not only enable easy deployment on various edge computing devices but also effectively meet the practical needs of real-time monitoring and precise identification in field environments. Through in-depth investigation of the impact mechanisms of data quality and quantity on model performance, this study not only provides important theoretical support for the development of agricultural artificial intelligence but also opens alternative technological pathways for locust and grasshopper classification and control. Looking ahead, we will advance research in the following directions: further expanding and optimizing dataset scale; deeply exploring the feature extraction mechanisms of deep networks for complex images; and continuing to optimize model architecture design based on the core value of lightweight networks in resource-constrained scenarios. By maintaining high accuracy while improving computational efficiency, we aim to develop more lightweight and precise intelligent recognition systems to meet the practical demands of diverse agricultural applications.

## Resource availability

### Lead contact

Further information and requests for resources should be directed to and will be fulfilled by the lead contact, Ning Wang (wangningis@163.com).

### Materials availability statements

This study did not generate new unique reagents.

### Data and code availability

• Data have been deposited at Mendeley data and are publicly available as of the date of publication. The dataset can be accessed at the following https://doi.org/10.17632/ynb99zjpxh.1.
• The code can be found at the URL: https://github.com/Fancy08zhen/Code.git.
• Any additional information needed to reanalyze the data reported in this paper is available from the lead contact upon request.

## Acknowledgments

We thank 10.13039/501100001809National Natural Science Foundation of China for funding (32160633), Innovative Research Team in Universities of Inner Mongolia Autonomous Region (NMGIRT2320), 10.13039/501100012166National Key R&D Program of China (2024YFC2607701-1, 2022YFD1401102).

## Author contributions

Conception and design, Y.Z. and N.W.; acquisition of data, Y.Z.; statistical analysis and interpretation, Y.Z., N.W., and Y.L.; drafting the article, Y.Z., H.H., H.Y., Y.S., W.W., N.W., and Y.L.; critically revising the article, Y.Z., H.H., H.Y., Y.S., W.W., N.W., and Y.L.; technical support, Y.Z.; study supervision, N.W. and Y.L.

## Declaration of interests

The authors declare no competing interests.

## STAR★Methods

### Key resources table

**Table:** 

REAGENT or RESOURCE	SOURCE	IDENTIFIER
**Deposited data**		

Code	This paper	https://github.com/Fancy08zhen/Code.git
Data	This paper	https://doi.org/10.17632/ynb99zjpxh.1

**Software and algorithms**		

Python 3.10.11	Python	https://www.python.org
PyTorch 1.10.0	PyTorch	https://pytorch.org

### Method details

#### Data collection

Our dataset for this study was compiled from three distinct sources. Firstly, we collected locust and grasshopper images from the iNaturalist and GBIF platforms. These platforms provide a wealth of image resources for global biodiversity, covering locust and grasshopper species from different regions and environments.^29^^,^^30^ Secondly, we conducted targeted data collection through searching engines included Baidu, Bing, and Google. In other words, using generic name related to locusts and grasshoppers, we gathered 525632 images from both public online resources and datasets provided by research institutions. Lastly, we obtained images of locust and grasshopper species photoed manually in Siziwang Banner, Inner Mongolia, China. These images provide valuable local and ecological diversity to the dataset.

The datasets cover 60 different locust and grasshopper genera, and although the dataset size is substantial, the quality varies due to differences in image size, resolution, and angle from which it was taken. Some images are clear and detailed, while others may appear blurry or poorly lit due to shooting restrictions. In addition, the locust and grasshopper images were taken in various environments, including deserts, grasslands, forests, wetlands, and laboratory settings. This diversity adds to the high complexity and diversity of the dataset, especially in terms of background, light conditions, and locust and grasshopper species.

To better facilitate model training and recognition tasks, we divided the dataset into two parts: dataset A and dataset B. Dataset A, which contains 14,156 raw images, is considered a smaller dataset and is primarily used for initial training and validation. Dataset B contains 68,807 raw images, which is relatively larger and suitable for more in-depth training and testing. As shown in Figure S1, the diversity and complexity of the datasets pose challenges for locust and grasshopper genus identification, but they also provide rich and diverse sample data for model training. This kind of diversity is essential for improving generalization ability and practical application value of the model.

#### Dataset preparation

To improve the data quality, the dataset underwent a thorough preprocessing procedure. Initially, the data were filtered to exclude blurry, duplicate, missing images, as well as those depicting nymphs, thereby ensuring the accuracy and effectiveness of the dataset. Subsequently, to standardize the images for input into the model, OpenCV (CV2) was used to resize all images to a uniform resolution of 224 × 224 pixels, guaranteeing consistency across all inputs.

Furthermore, to enhance the robustness of the model, mitigate the risks of overfitting, and improve its generalization performance on unseen data, data augmentation techniques were implemented using Keras. Specifically, the augmentation methods encompassed random rotation, horizontal and vertical shifts, counterclockwise shear transformation, random scaling factors, and random flipping. As shown in Figure S2, these techniques diversified the training data by applying diverse transformations to the images, allowing the model to adapt to various conditions. Consequently, this improved its capacity to generalize across numerous environments and situations.^31^

Following the preprocessing and augmentation operations, the experimental datasets were significantly expanded. Dataset A increased from 14,156 images to 60,000 images, while Dataset B grew from 68,807 images to 240,000 images. To guarantee the efficacy of the training process and the reliability of the validation results, Dataset A was divided into three subsets: a Training Set (60%), a Validation Set (20%), and a Test Set (20%). Similarly, Dataset B was divided into a Training Set (80%), a Validation Set (10%), and a Test Set (10%). This division ensured diverse model training and fair evaluation, thereby further enhancing the credibility of the experimental results.

#### EfficientNet

The EfficientNet utilized in the research is a convolutional neural network (CNN) architecture introduced by Google researchers Tan and Le (2019). The central concept of EfficientNet is to systematically scale the network’s width, depth, and image resolution using a method known as Compound Scaling.^32^^,^^33^ As opposed to traditional manual adjustments and scaling, EfficientNet attains superior performance and efficiency by balancing the various dimensions of the network through a straightforward yet effective scaling rule. The network comprises stacked convolutional modules, as shown in the EfficientNet Network Architecture. The initial layer is a regular 3 × 3 convolutional kernel with a stride of 2, which includes a Batch Normalization (BN) layer and the Swish activation function. The middle section consists of 16 convolutional modules; while the final segment comprises a regular 1 × 1 convolutional layer (including BN and the Swish activation function), an average pooling layer, and a fully connected layer.

**Table:** EfficientNet Network Architecture

Stage	Operator	Resolution	Layers	Channels
1	Conv3x3	224 × 224	1	32
2	MBConv1, k3x3	112 × 112	1	16
3	MBConv6, k3x3	112 × 112	2	24
4	MBConv6, k5x5	56 × 56	2	40
5	MBConv6, k3x3	28 × 28	3	80
6	MBConv6, k5x5	28 × 28	3	112
7	MBConv6, k5x5	14 × 14	4	192
8	MBConv6, k3x3	7 × 7	1	320
9	Conv1x1& Pooling &FC	7 × 7	1	1280

A detailed description of the EfficientNet model is presented in Table

#### MBConv and GhostConv

MBConv(Mobile Inverted Residual Bottleneck)is the cornerstone of the EfficientNet architecture, designed to maximize computational efficiency and model performance. The structure of the MBConv module primarily consists of the following components: Firstly, it employs depthwise separable convolution to decompose standard convolution into depthwise convolution and pointwise convolution, drastically reducing the number of parameters and computational overhead, making it particularly suited for mobile devices. Secondly, MBConv adopts a linear bottleneck design that involves first decreasing the number of channels, then extracting features through depthwise convolution, and finally restoring the number of channels using 1 × 1 convolution. This method significantly boosts model performance. In addition, MBConv provides optional residual connections to help retain input information and enhance learning capabilities. This efficient and adaptable structure enables EfficientNet to maintain a low computational resource consumption while achieving exceptional classification performance and outstanding results in various computer vision tasks.^34^. (Refer to Figure S3A for an illustration)

GhostConv is an innovative convolution operation designed to minimize the computational and parametric complexity of convolutional neural networks (CNNs) while perserving robust feature representation capabilities. The fundamental concept behind GhostConv is to split standard convolution into two stages. In the first stage, a smaller number of filters are utilized to perform standard convolution for feature extraction. In the second stage, additional “ghost” feature maps are generated through straightforward linear operations, such as pointwise convolution or low-rank decomposition. This two-stage process enables the network to produce a richer set of features while drastically cutting down on computational resource consumption.^35^ The main advantages of GhostConv lie in its efficiency and flexibility. It improves the expression ability while maintaining a low computational footprint. This makes GhostConv an excellent choice for mobile devices and environments with limited resources. By leveraging this approach, CNNs can achieve superior performance without the usual computational overhead, making them more practical for real-world applications. (Refer to Figure S3B for a visual representation.)

#### Channel wise and pixel wise attention

The attention mechanism is a computational method that mimics human visual attention. It aims to enhance the feature representation ability of neural networks when processing complex tasks. This mechanism dynamically assigns different weights to input features, emphasizing important information while suppressing redundant or irrelevant information. In deep learning, attention mechanisms are widely used in fields such as natural language processing and computer vision. They are mainly divided into two categories: channel attention and spatial attention. Channel attention focuses on the importance of different channels in the feature map, highlighting key channels through weighting; Spatial attention focuses on different positions in the feature map, emphasizing important regions. By combining these two attention mechanisms, information can be captured and utilized more comprehensively. This significantly improving the performance of the model, especially in tasks such as object detection, image classification, and machine translation.

The Channel-wise Principal Component Attention (CPCA) attention mechanism is a feature enhancement method that combines channel and spatial attention. Its aim is to improve the performance of convolutional neural networks.^36^ Specifically, the CPCA parsing process is as follows: The dimensions of the input feature map are *H* × *W*, with *C* channels (i.e., the dimension of the input channels). The shape of the input feature map is defined as follows: *C* × *H* × *W*, with each channel representing an *H* × *W* two-dimensional matrix, and all the channels are stacked together to form a three-dimensional tensor. The process of spreading each channel entails the execution of a spreading operation for each channel *i* (*i* = 1, 2, …, *C*), with the objective of disseminating the *H* × *W* 2D matrix into a 1D vector.: $Flattened\;channel_{i}=reshape\left(channel_{i}\right)\;shape=\left(H\times W\right)$.Performing this operation for all *C* channels yields a matrix of shape *C* × (*H* × *W*):

(Equation 1) $$X=\left[\begin{array}{c} Flattened\;channel_{1}\\ \begin{array}{c} Flattened\;channel_{2}\\ \vdots \end{array}\\ Flattened\;channel_{C} \end{array}\right]$$

The covariance is calculated for the matrix *X*. The covariance matrix reflects the correlation between different channels. For each pair of channels *i* and *j*, the elements in the covariance matrix represent the covariance between channel *i* and channel *j*:

(Equation 2) $$C^{*}=\frac{1}{H\times W-1}X^{T}X$$

Where *C* ∗ is a *C* × *C* covariance matrix representing the correlation between channels.

Principal Component Analysis (PCA) is performed on the covariance matrix *C*∗. PCA is performed by eigenvalue decomposition of the covariance matrix *C*∗*vi* = *λivi*, where *λi* is the *i*th eigenvalue and *vi* is the corresponding eigenvector. Find the principal components of the data (i.e., the direction of the maximum variance). Selecting the top *k* principal components: the top *k* largest eigenvalues and their corresponding eigenvectors are selected by arranging the eigenvalues in descending order. Channel compression is performed by projecting the raw data of each channel onto the first *k* principal components:

(Equation 3) $${X\;}_{\text{compressed}}=X\cdot V_{k}$$

where, *Vk* is the matrix containing the first *k* principal components. In this way, the original *C* channels are compressed into *k* new channels and these *k* channels contain most of the useful information. The new feature map is obtained by reconstructing using the compressed channels. The number of channels of the new feature map is *k* and the computation and memory consumption are reduced with guaranteed information.

This mechanism is flexible and can be combined with various convolutional network architectures to significantly improve the performance of the model, especially in tasks such as object detection and image classification. (Figure S4)

The Efficient Channel Attention (ECA) attention mechanism first extracts global information from the channels of the input feature map using global average pooling. Then, it applies a one-dimensional convolution with an adaptive kernel size to learn the weights of each channel. The kernel size is usually set to the square root of the number of channels to reduce the number of parameters and maintain efficiency. Subsequently, the output of the convolution is transformed into weights for each channel through an activation function such as Sigmoid, reflecting the importance of each channel. These weights are then multiplied by the corresponding channels of the original feature map to enhance important features and suppress unimportant ones. This achieves effective channel attention focusing.^37^ In summary, the ECA mechanism improves computational efficiency by using an adaptive kernel size and reducing the number of parameters. At the same time, it effectively enhances important channel features and suppresses irrelevant ones, thereby boosting the model’s performance.^38^

#### Improved model

In order to create a lighter and more accurate model, we made several improvements as shown in Figure S5. Our modifications were based on EfficientNet, where we replaced the MBConv in the convolutional modules with GhostConv. This was done to maintain good feature extraction capabilities while reducing the computational complexity of the network, which also led to a reduction in model size. Furthermore, to enhance the model’s ability to highlight important features, we replaced the SE attention mechanism in GhostConv with the ECA attention mechanism. This made the module more lightweight and improved its convolutional capabilities. Finally, we added a CPCA attention mechanism before the convolution, which significantly enhanced the performance of the entire model. To emphasize its modifications and facilitate writing, we abbreviated this improved model as CGENet.

#### Transfer learning

Transfer learning is an innovative approach in machine learning where the primary goal is to leverage knowledge gained from one domain to improve the learning efficiency and performance of a model on a new, related task. This approach is especially advantageous in scenarios where labeled data for the new task is scarce or where training costs are high. By minimizing the reliance on extensive training data for the new task, transfer learning expedites the learning process and significantly enhances the model’s performance. The process of transfer learning typically involves two main stages: pre-training and fine-tuning.

In the pre-training stage, the model is trained on a large-scale dataset to learn general feature representations. These features, such as edges, corners, and textures in images, are widely applicable and can generalize to multiple tasks. The goal of this stage is to capture fundamental features that are useful for various image-related tasks. During the fine-tuning stage, the pre-trained model undergoes adjustments on a specific dataset for the target task. This process optimizes the network parameters to better adapt to the unique requirements of the new task. By leveraging the knowledge learned during pre-training, transfer learning significantly reduces the need for large amounts of labeled data for the new task.

The advantage of transfer learning is its ability to leverage the knowledge learned from the pre-trained model. It reduces the reliance on extensive training data, shortens training time, and enhances the model’s accuracy on the new task. This approach is particularly beneficial in cases where labeled data are limited or training resources are insufficient. By enabling models to learn from related tasks and adapt to new ones more efficiently, transfer learning demonstrates its strong potential in various machine learning applications.

#### Equipment environment and parameter settings

In this experiment, we are utilizing a Python 3.10.11 environment, along with the PyTorch 1.10.0 deep learning framework to construct and improve our models. The hardware specifications include a CPU, which is an AMD Ryzen 7 6800H with Radeon Graphics running at 3.20 GHz, and 16GB of memory. The GPU is an NVIDIA GeForce RTX 3060. The initial parameter settings for the model are as follows: a learning rate of 0.001, 100 epochs, a batch size of 16, and the SGD optimization algorithm. Furthermore, we have employed a Lambda function to dynamically adjust the learning rate of the optimizer. The purpose of this dynamic adjustment is to progressively decrease the learning rate, thereby aiding the algorithm in converging more stably during the later stages of training. This approach helps to prevent premature termination or oscillation.

#### Model evaluation indicators

The study employs Accuracy(Acc), Precision(Prec), Recall(Rec), and F1-score(F1) as evaluation metrics for the classification model. The specific calculation methods for these metrics are as follows:

(Equation 4) $$A\text{cc}uracy\left(Acc\right)=\frac{TP+TN}{TP+FP+TN+FN}$$

(Equation 5) $$Precision\left(Prec\right)=\frac{\text{TP}}{\text{TP}+\text{FP}}$$

(Equation 6) $$R\text{ecall}\left(R\text{ec}\right)=\frac{TP}{TP+FN}$$

(Equation 7) $$F\text{measure}\left(F1\right)=2\times \frac{Precision\times R\text{ecall}}{Precision+R\text{ecall}}$$

TP (True Positive) refers to the number of instances that are actually consistent with the classification and are correctly identified by the model; FP (False Positive) refers to the number of instances that are actually inconsistent with the classification but are incorrectly identified by the model as belonging to a certain category; TN (True Negative) refers to the number of instances that are actually consistent with the classification and are correctly identified by the model as not belonging to a specific category; FN (False Negative) refers to the number of instances that are actually consistent with the classification but are incorrectly identified by the model as not belonging to the same category.

### Quantification and statistical analysis

In this study, the PyTorch framework was used to construct all deep learning models, and the training and testing process of the models was completed in the Python environment. We used multidimensional evaluation metrics: Accuracy, Precision, Recall, and F1-score were used for classification performance; FLOPs and Params were examined for computational efficiency. These metrics are systematically presented in Tables 1, 2, 3, and 4 and in Figures 1, 7, and 8.

In particular, in the experiments of Figure 4, we use a 5-fold cross-validation approach and quantify the performance differences between models by *p*-values and ∗∗∗ (denotes *p* < 0.001). In addition, to verify the practical value of the model, we developed a mobile application based on Android Studio and conducted performance tests on real mobile devices, and the relevant results are displayed in Figure 5.
