# Supplementary material for: Design of a lightweight recognition network for adult locusts and grasshoppers based on deep learning
Source: iScience. 2025 Jul 10;28(8):113096. doi: 10.1016/j.isci.2025.113096 (PMC12361779; doi:10.1016/j.isci.2025.113096)
Supplement: Document S1. Figures S1–S5 [file mmc1.pdf]

iScience, Volume *28*

Supplemental information

Design of a lightweight recognition network for adult locusts and grasshoppers based on deep learning

Youchen Zhen, Haibin Han, Hongru Yue, Yanmin Shan, Wei Wu, Ning Wang, and Yanyan Li

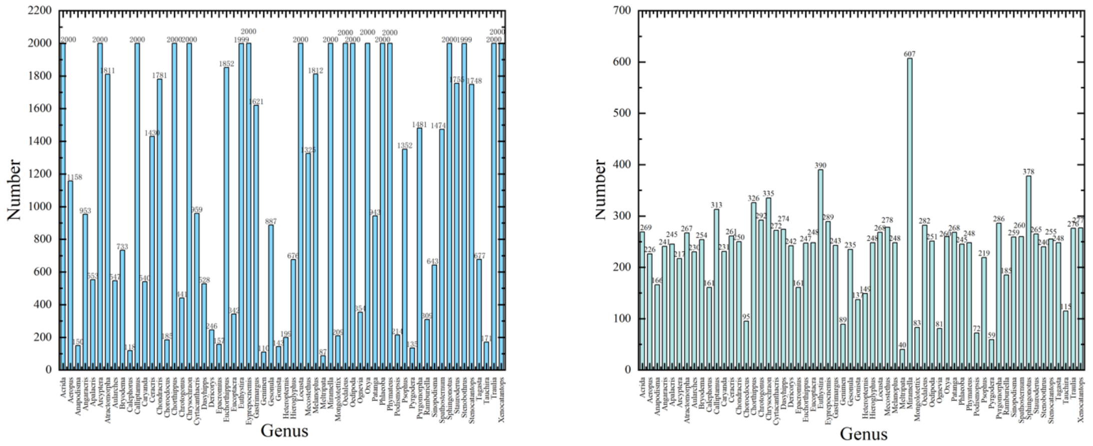

(A) (B)

Figure S1. Datasets details

Figures (A) and (B) show the original images of locusts and grasshoppers genera in both datasets 60

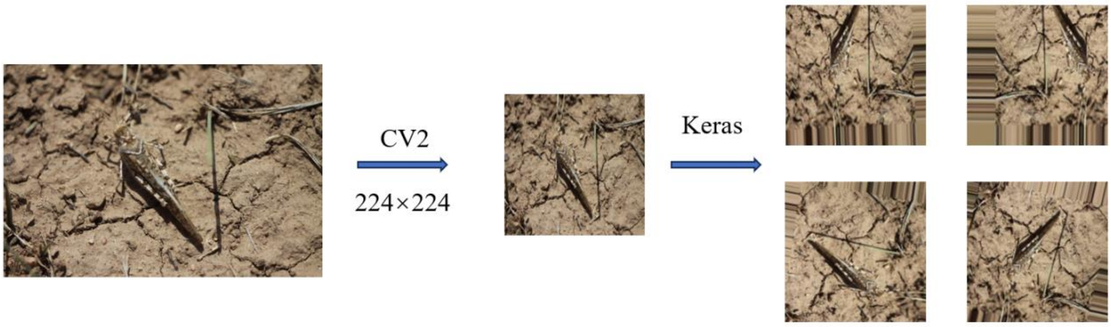

Figure S2. Image preprocessing

The figure illustrates the two-part preprocessing of image data through resizing and quantity augmentation

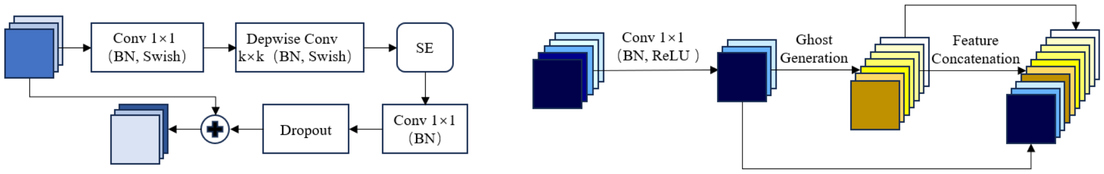

(A) (B)

Figure S3. Two types of convolution modules

(A) shows the structure of MBConv (B) shows the structure of GhostConv.

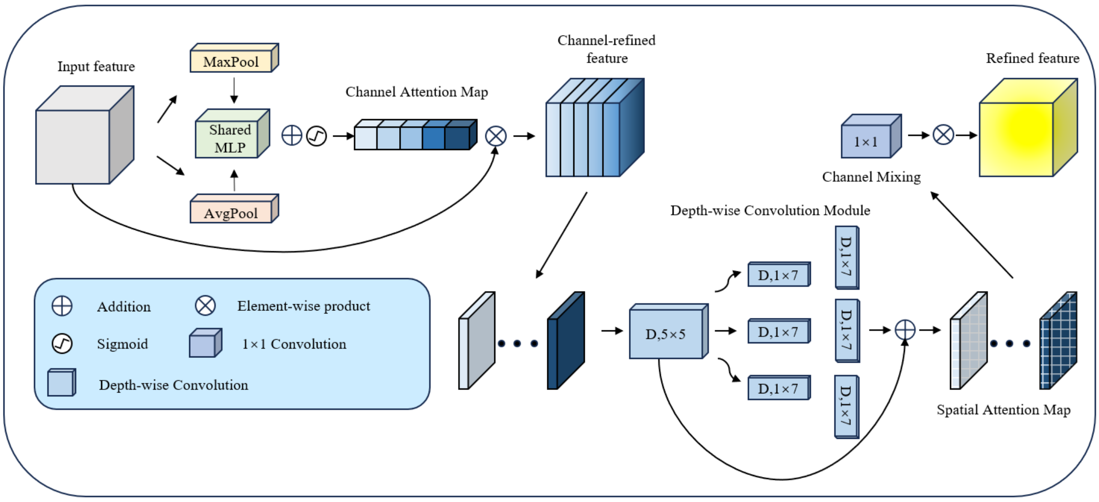

Figure S4. Channel-wise Principal Component Attention

The figure shows the detailed structure and image processing of Channel-wise Principal Component Attention

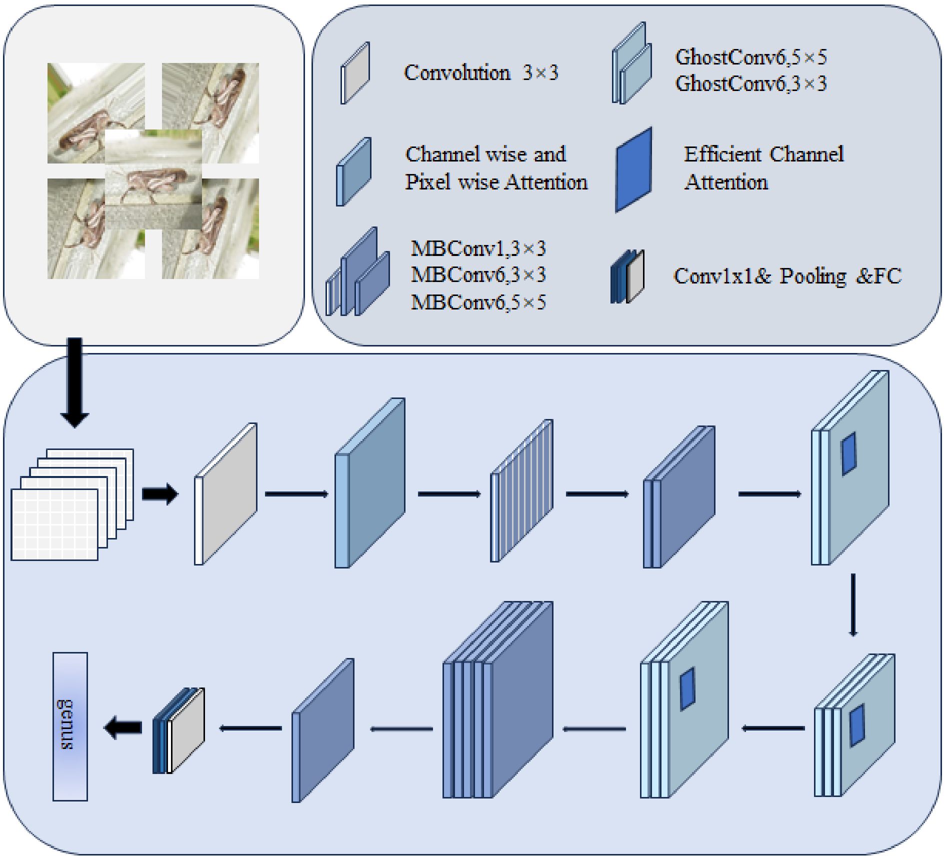

Figure S5. Our network model structure

The figure shows the detailed structure of CGENet and the image processing process
